# Food Sources of Sodium Intake in an Adult Mexican Population: A Sub-Analysis of the SALMEX Study

**DOI:** 10.3390/nu9080810

**Published:** 2017-07-27

**Authors:** Eloisa Colin-Ramirez, Ángeles Espinosa-Cuevas, Paola Vanessa Miranda-Alatriste, Verónica Ivette Tovar-Villegas, JoAnne Arcand, Ricardo Correa-Rotter

**Affiliations:** 1 Sociomedical Research Department, Instituto Nacional de Cardiología ‘Ignacio Chávez’, Mexico City 14080, Mexico; veronicatovar92@hotmail.com; 2 Consejo Nacional de Ciencia y Tecnología (CONACYT), Mexico City 03940, Mexico; 3 Nephrology and Mineral Metabolism Department, Instituto Nacional de Ciencias Médicas y Nutrición Salvador Zubirán, Mexico City 14080, Mexico; angespinosac@gmail.com (Á.E.-C.); pvma2000@hotmail.com (P.V.M.-A.); correarotter@gmail.com (R.C.-R.); 4 Faculty of Health Sciences, University of Ontario Institute of Technology, Oshawa, ON L1H 7K4, Canada; joanne.arcand@uoit.ca

**Keywords:** salt, hypertension, processed foods

## Abstract

Excessive dietary sodium intake increases blood pressure and cardiovascular risk. In Western diets, the majority of dietary sodium comes from packaged and prepared foods (≈75%); however, in Mexico there is no available data on the main food sources of dietary sodium. The main objective of this study was to identify and characterize the major food sources of dietary sodium in a sample of the Mexican Salt and Mexico (SALMEX) cohort. Adult male and female participants of the SALMEX study who provided a complete and valid three-day food record during the baseline visit were included. Overall, 950 participants (mean age 38.6 ± 10.7 years) were analyzed to determine the total sodium contributed by the main food sources of sodium identified. Mean daily sodium intake estimated by three-day food records and 24-h urinary sodium excretion was 2647.2 ± 976.9 mg/day and 3497.2 ± 1393.0, in the overall population, respectively. Processed meat was the main contributor to daily sodium intake, representing 8% of total sodium intake per capita as measured by three-day food records. When savory bread (8%) and sweet bakery goods (8%) were considered together as bread products, these were the major contributor to daily sodium intake, accounting for the 16% of total sodium intake, followed by processed meat (8%), natural cheeses (5%), and tacos (5%). These results highlight the need for public health policies focused on reducing the sodium content of processed food in Mexico.

## 1. Introduction

Excessive dietary sodium intake increases blood pressure [1] and increases risk of hypertension, cardiovascular disease, stroke and chronic kidney disease [2,3,4,5,6,7]. Globally, it is estimated that 4.1 million deaths and 83 million years of disability were attributable to an excess dietary sodium intake in 2015 [8]. In Mexico, the prevalence of hypertension in those 20 years and older was 32.3% in men and 30.7% in women in 2012 [9]. Recently, mean dietary sodium intake in a healthy adult Mexican population was estimated to be 3150 mg/day (95% confidence interval: 3054, 3246 mg/day), being as high as 3735 mg/day among men, as measured by 24-h urinary sodium excretion [10]. These data show that sodium intake in Mexican population is higher than the World Health Organization (WHO) recommended intake of less than 2000 mg/sodium per day, a level set to reduce blood pressure and cardiovascular risk at the population level [11]. Together, these data highlight the need for population-based strategies in Mexico to achieve the WHO global target of reducing dietary sodium intake by 30% by 2025, for the prevention and control of non-communicable diseases [12].

Major sources of dietary sodium vary among different regions. In Western diets, the majority of dietary sodium comes from packaged and prepared foods (≈75%), with a small contribution from discretionary salt that is added in cooking or at the table [13]. Conversely, in countries such as India [14], China [15] and Japan [16], discretionary use of salt remains a major source of dietary sodium. For example, in a Chinese population, it was found that 76% of dietary sodium came from salt added in home cooking, with a small contribution of processed foods [17]. In the Latin America region, Argentina reported that packaged and processed foods contributed between 65% and 70% to daily sodium intake [18]. In Mexico, there is no available data on the main food sources of dietary sodium. Knowledge of this information is key to identify targets and inform population-based strategies for sodium reduction at the population level. Thus, the main objective of this study was to identify and characterize the major food sources of dietary sodium in a sample of the Mexican Salt and Mexico (SALMEX) cohort. We also aimed to quantify the percentage of total sodium intake coming from different types of food, based on an analysis of food categories.

## 2. Materials and Methods 

### 2.1. Study Population 

This cross-sectional analysis was based on data from the Salt and Mexico (SALMEX) study. SALMEX was a cross-sectional study aimed at assessing the average salt, potassium, and iodine intake in an adult workers cohort from the National Institute of Medical Sciences and Nutrition Salvador Zubirán (INCMNSZ), in Mexico City, Mexico. One thousand and nine men and women between 18 and 65 years old were recruited in this cohort between 2010 and 2011. Participants of the SALMEX study were recruited from the INCMNSZ via an informative session on sodium intake and its role in human health that was delivered to the personnel from all the different areas and departments of this institution. At the end of these sessions, the SALMEX study was introduced to the attendees, who were invited to participate. Exclusion criteria were: history of heart failure, advanced kidney or liver disease, intestinal resection, diuretic drug initiation during the previous five days to enrolment, active infection, pregnancy, and lactation.

All subjects gave their informed consent for inclusion before they participated in the SALMEX study. The study was conducted in accordance with the Declaration of Helsinki, and the protocol was approved by the Health Research and Ethics Boards of the Instituto Nacional de Ciencias Médicas y Nutrición Salvador Zubirán (INCMNSZ) (REF. 191). Full results of the SALMEX study are currently being revised to be considered for publication, and have been published only as a conference abstract [19].

For the purposes of this sub-analysis of the SALMEX study, only participants with a complete and valid three-day food record were included.

### 2.2. Assessments

#### 2.2.1. Anthropometric and Blood Pressure Measurements

Body weight and height were measured according to standard protocols [20]. Subjects wore light clothes and were barefoot. Body mass index (BMI) was calculated by dividing total body weight (kilograms) by height squared (square meters). Systolic and diastolic blood pressure were measured three times on the right arm, with the participants in a seated position and after a five-minute rest, using an Omron HEM-907XL automated sphygmomanometer (Omron Health Care, Inc., Vernon Hill, IL, USA).

#### 2.2.2. Estimating Total Sodium Intake

As part of the SALMEX cohort, all included participants completed a three-day food record to estimate dietary sodium intake. Trained dietitians provided detailed instructions to the participants on how to fill out the food diaries. Subjects were asked to record all food and beverages consumed during the three days prior to the study visit, using standard household measures (e.g., cups and tablespoons) or commercial measures (e.g., weight of commercially packaged foods as given on the label or number of pieces consumed). All food records were reviewed by the study dietitian during an interview with the patient to identify any missing food items and to clarify food item descriptions and portion sizes using standardized food models.

Food records were analyzed by trained personnel to estimate total sodium intake by using a nutrient software program (Nutrikal^®^VO, V2, Consinfo, S.C., Mexico City, Mexico), which contains the nutritional composition of common Mexican foods. Additional food items were added to the Nutrikal database when none of the food items contained in the current database reflected the actual food consumed by the patient, or when sodium content for that specific food item was not reported in the database (e.g., panela cheese, some seasonings and sauces, etc.). Sodium content for food items added to the database was obtained from food labels or the Tables of Composition of Mexican Foods and Food Products [21]. A mean dietary intake from the three days was estimated for energy (kcal/day) and sodium (mg/day). Table salt was not considered for food record analysis due to the complexity of accurately estimating the amount of salt added in cooking or at the table, either at home or restaurants. Generic restaurant meals included in the Nutrikal database such as pizzas and hamburgers were considered for food record analysis; however, in the case of more specific restaurant meals not included in the database, such as meat or pasta dishes and salads, for which nutritional information was not available, ingredients, except for salt and salty seasonings, were considered separately for food record analysis.

Only participants with a complete and valid three-day food record were considered for analysis. A complete food record was defined as that with three days recorded; whilst a valid food record was considered that with a plausible energy intake reported (defined as ≥500 kcal/day and ≤4000 kcal/day) [22,23,24].

#### 2.2.3. Identifying Food Sources of Sodium

Because the nutrient software program employed for food record analysis did not provide a break-down of dietary intake information to individual food items entered for analysis, and only provided the overall dietary intake information from the whole day or from the total amount of days recorded (e.g., total amount of sodium provided by all the foods entered from the three days), it was not feasible to sum up total sodium from all foods recorded within the food category to identify the main food sources of sodium, and thus we followed a food record-searching approach to search for relevant food sources of sodium in the food records. For this purpose, relevant food sources of sodium were identified prior to food record searching according to the following criteria:

(a) To identify foods in the food supply that contain high amounts of sodium, the Nutrikal^®^VO (V2) nutrient software database, which contains the nutritional composition of common Mexican foods, was used. Database food items with a sodium content ≥480 mg/100 g of food product were identified. The cut-off of 480 mg of sodium/100 g of product as a means to identify potential food sources of sodium, was based on the rationale that a percent daily value (%DV) of 20% provided by a product-specific serving size is considered high by the U.S. Food and Drug Administration. Since the reference daily value was 2400 mg/sodium, 480 mg represented this 20% DV [25]. In Mexico, there is currently no a reference %DV to classify nutrient DVs reported on the food labels as high or low. Additionally, in this study, due to the complexity of determining product-specific serving sizes, we selected a standard portion of 100 g of product for all food items to identify a sodium content of 480 mg.
(b) To identify food sources of sodium not included in the Nutrikal^®^VO (V2) nutrient software database, an expert panel made up of two nutritional epidemiologists and one nutritionist scientist reviewed the Nutrikal^®^VO (V2) food list and developed a list of foods that were missing in the database, but are highly consumed by the Mexican population. The additional foods considered for inclusion were confirmed to have a sodium content ≥480 mg/100 g (e.g., carnitas and cecina tacos, panela cheese, chorizo, and some hot sauces and hot chili powder seasonings). Sodium content was obtained from food labels for packaged foods, and from the Tables of Composition of Mexican Foods and Food Products [21] in the case of tacos.
(c) To identify foods containing moderate amounts of sodium that are consumed highly frequently by the Mexican population, and thus cumulatively may contribute significant amounts of sodium, the same expert panel identified food items with sodium content >120 mg sodium/100 g of product. At this stage, foods such as Mexican street food [Mexican little whims (antojitos mexicanos)], cereal bars, some packaged cookies and bakery goods, and some type of chips, were included. Sodium content was obtained from the Nutrikal^®^VO (V2) nutrient software database, food labels, and the Tables of Composition of Mexican Foods and Food Products [21]. Based on the rationale that a 5% DV or less (equivalent to 120 mg/sodium or less according to a DV of 2400 mg/sodium) provided by a product-specific serving size is considered low [25], we also focused on products with sodium content >120 mg/100 g of product in order to ensure inclusion of moderate-sodium food items that may represent a relevant food source of sodium in this population.

All additional food items considered for inclusion as described in points b and c, were also considered for food record analysis to estimate total sodium intake. After food sources of sodium were identified (as previously described), these were intentionally searched in the food records by trained dietitians during the food record searching phase. The amount of sodium in mg provided by the total amount of each specific food item consumed per person during the three days and an average from the three days was recorded.

Similar to the food record analysis for estimating total sodium intake, and due to the complexity of accurately estimating the amount used, table salt was not considered during the food record searching phase, and in the case of specific restaurant meals not included in the database and for which there was no nutritional information available, such as meat or pasta dishes and salads, ingredients, except salt and salty seasonings, were considered separately. After the food record searching phase was completed, all food items (sources of sodium) identified in the food records were classified into 33 sodium-focused food categories based on Health Canada’s Guidance for the Food Industry on Reducing Sodium in Processed Foods [26], and adapted for Mexican food, considering the raw material used for their elaboration, similar nutritional content, and culinary practices used.

#### 2.2.4. Twenty-Four-Hour Urinary Sodium Excretion

One 24-h urine sample was collected from each participant during the last day of food recording. Participants were provided with detailed verbal and written instructions on how to collect the urine sample. They were asked to discard the first morning void and to collect all urine over the following 24 h, including the first void on the next morning. Participants were given a preservative-free container to collect the urine sample. Urinary sodium was determined by using the ion selective electrode method [27], and urinary creatinine was measured by Jaffe’s colorimetric assay in automated analyzers (Synchron Cx5 PRO autoanalyser, Beckman Coulter Inc., Fullerton, CA, USA). Completeness of the 24-h urine samples was determined based on creatinine excretion by dividing total urinary creatinine by body weight in kilograms. Participants with urinary creatinine levels within the standard creatinine excretion rates (15–25 mg/kg/24-h for men and 10–20 mg/kg/24-h for women) [28] were considered for estimating 24-h urinary sodium excretion.

### 2.3. Statistical Analysis 

Continuous variables were expressed as mean ± standard deviation, and categorical variables were presented as absolute (number of participants) and relative frequencies (percentages). For comparison of continuous variables, the Student’s *t*-test for independent samples was employed, whilst the Pearson chi-square test or Fisher’s exact test were used for categorical variables.

For each individual, the following parameters related to the sodium-focused food categories were estimated:

(a) an average daily sodium intake in mg from the three days recorded, provided by each food category; and
(b) the percentage that each food category contributed to total sodium intake, which was estimated by the three-day food record.

Since only one 24-h urine sample was collected and food sources of sodium were obtained from three days, percent of contribution to total sodium intake provided by each food category was based on total sodium intake estimated by the three-day food record instead of that estimated by the 24-h urinary sodium excretion.

The percent of the study population consuming at least one of the food items from each sodium-focused food category on at least one of the three days recorded was estimated, and it was considered the proportion of consumers for each food category. We conducted analyses for the entire study population, and then analyses considering only consumers of each food category.

Means [95% confidence intervals (CI)] in the entire study population (per capita) and among consumers were estimated for the two parameters listed above for all food categories, except for those with an overall consumer prevalence less than 5%, since estimates may be less precise in such small populations.

## 3. Results

Of the 1009 participants recruited in the SALMEX study, 979 provided a complete three-day food record, of which 950 were valid. Of these, 698 provided a complete 24-h urine sample. Overall, 950 participants with a complete and valid three-day food record were included for analysis of food sources of sodium, while only those with a complete 24-h urine sample (*n* = 698) were considered for estimating 24-h urinary sodium excretion. Characteristics of the study population by sex are shown in Table 1. Women were older and had lower systolic and diastolic blood pressure compared to men. Dietary sodium intake was 2647.2 ± 976.9 mg/day in the entire study population, observing a higher consumption among men (3018.2 ± 1091.9 mg/day) compared to women (2422.9 ± 823.6, *p* < 0.001), as measured by three-day food records. Twenty-four-hour urinary sodium excretion showed the same trend, with a higher excretion in men (4167.7 ± 1520.2 mg/day) than in women (3118.3 ± 1156.4, *p* < 0.001)

Proportion of consumers for each sodium-focused group is shown in Table 2. In the overall population, savory bread (84%), processed meat (73%), natural cheeses (70%), sweet bakery goods (68%), salad dressings and mayonnaise (48%), cookies and cereal bars (39%), chips (28%), tacos (24%), breakfast cereal (22%), and canned peppers (19%), were the 10 food groups with the highest proportion of consumers; whilst chicken nuggets (2%), instant soups (1%), chocolate milk powder (1%), olives (0.7%), powdered milk (0.6%), hot cakes flour mix (0.6%), and peanut butter (0.1%), were the food groups with less than 5% of consumers. Additionally, natural cheeses (75% vs. 64%, *p* < 0.001), breakfast cereal (25% vs. 17%, *p* = 0.004), hot sauces and chamoy (bottled snack sauces) (11% vs. 7%, *p* = 0.04), and hot chili powder seasonings (7% vs. 3%, *p* = 0.004) showed a higher proportion of consumers among women compared to men; contrarily, there was a higher percentage of consumers of tacos (30% vs. 20%, *p* = 0.001) and hamburgers (9% vs. 5%, *p* = 0.03) in men than women.

Table 3 shows the total sodium contributed in mg by the top 26 food categories based on the prevalence of consumers for each food category, excluding those with a proportion of consumers less than 5%. Processed meat (223 mg/day), savory bread (209 mg/day), sweet bakery goods (178 mg/day), natural cheeses (118 mg/day) and tacos (114 mg/day) were the five leading categories in the overall population. This table also shows the percent of total sodium intake estimated by three-day food records attributed to each category; dietary sodium provided by these top 26 food categories accounted for the 52.7% of total sodium intake per person in this population, with bread products (savory bread and sweet bakery) representing a 16% of total sodium intake, followed by processed meat (8%), natural cheeses (5%) and tacos (5%).

When only individuals who consumed the foods (consumers) were included in the analysis, pizza (582 mg/day), tacos (480 mg/day), seasonings (356 mg/day), hot chili powder seasonings (350 mg/day), and hamburgers (340 mg/day) were identified as the top five contributors to daily sodium intake. Tacos provided nearly 22% of total sodium intake among tacos consumers, followed by pizza that represented a 21% of total sodium intake among tacos consumers (Table 4).

**Figure 1:**
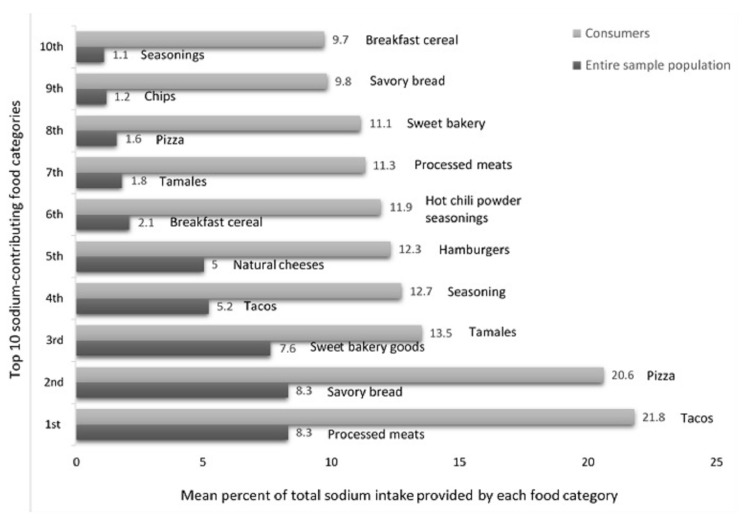
Top ten sodium-contributing food categories according to the percent of total sodium intake (as measured by three-day food records) attribute to each food category in the entire sample population (per capita) and among individuals who consumed the foods (consumers).

The top ten sodium-contributing food categories according to the percent of total sodium intake, as measured by three-day food records, attributed to each food category in the entire sample population (per capita) and among consumers, are summarizes in Figure 1.

## 4. Discussion

This is the first study aimed at identifying major food sources of sodium in a sample of the Mexican population. Of the 33 sodium-focused food categories identified in the diet of this population, processed meat was the main contributor to daily sodium intake, representing 8% of total sodium intake per capita. However, if savory bread (8%) and sweet bakery (8%) are considered together as bread products, these were the major contributor to daily sodium intake accounting for the 16% of total sodium intake. Similar results have been reported across diverse occidental countries such as Costa Rica (48%, reported as cereal and cereal products among women) [29], United Kingdom (34.6%) [17], Colombia (30.5%) [30], France (24.2%) [31], and Canada (13.9%) [32], where bread products were found to be the major contributors for daily sodium intake, although the percentage of contribution varies across these countries. In the United States (19.5%, including breads, grains and cereals) [17] and Brazil (between 10% and 11% across age groups) [33], this food category was the second and third main source of total sodium intake, respectively. Thus, recognizing that bread products are relevant sources of dietary sodium in the western diet, some countries in the Americas region have set voluntary or regulated targets and timelines for reducing sodium content in bread, among other food products, as part of an initiative proposed by the Pan American Health Organization in 2013 to achieve the WHO global target of a 30% relative reduction in salt intake by 2025 [34]. For example, Argentina, where bread accounts for almost 25% of total salt in the diet [18], has achieved a 25% average reduction in sodium content of bread from 2011 to 2013 [35]. Similarly, Chile has reported an average decrease in sodium levels in bread from >830 mg/100 g to 479 mg/100 g [35]. Likewise, Mexico in 2012 set its voluntary food reformulation initiative with a bread category including sliced bread and bolillo (a product similar to baguette) to reduce their sodium content by 10% in five years. Average baseline sodium levels of these products were estimated at 520 mg/100 g [36]. Currently, monitoring studies of this initiative in Mexico have not been reported. Results of this study highlight the need of a collaborative effort to reduce the sodium content of processed foods, not only bread, but also processed meat, cheese and cereal products that were identified as relevant food sources of sodium in this population. Importantly, natural cheeses were listed in the top 10 sodium-contributing food categories, while processed chesses were not; this may be explained by the high prevalence of consumers of natural cheeses (70%) in this population, highlighting the need for initiatives to reduce the sodium content in these type of cheeses. Also, it is important to implement a monitoring plan to evaluate adherence to these initiatives.

In Asian countries, salt and salty condiments remain the main source of dietary sodium intake. Data from the INTERMAP Study revealed that in the Chinese population, most (76%) dietary sodium was from salt added in home cooking, followed by soy sauce (6%); while in the Japanese sample of this same study, the main food source of dietary sodium was soy sauce (20%) [17]. In a more recent study in Japanese population, it was reported that the contribution to daily sodium intake of seasonings such as salt or soya sauce may be as high as 62% in men and 63% in women [16]. 

In this Mexican population, within the combined contribution for total sodium intake of seasonings (1.1%), mole (1.0%), hot chili powder seasonings (0.7%), and hot sauces and chamoy (bottled snack sauces) (0.5%) represented 3.3% of the total sodium intake per person. However, estimation of sodium intake from table salt added in cooking at home or outside home was not feasible, due to the challenges of self-reporting the amount of salt used for cooking, especially if somebody else cooked; however, we believe that table salt added to meals in cooking represents an important contribution to total sodium intake, since home cooking remains a common practice and nearly 10% of Mexican population have their afternoon meal at local kitchens that serve homemade-style meals [37]. Importantly, seasonings and hot chili powdered seasonings represented 13% and 12% of total sodium intake, respectively, among consumers, highlighting the relevance of making people aware of the importance of reducing not only the use of table salt, but also salty seasonings, for the purpose of reducing dietary sodium intake, by providing alternatives to enhance the flavor of the food without adding salt or salty seasonings.

Additionally, in Mexico there is limited information on the nutritional content of restaurant foods, especially for sit-down restaurants, due to the lack of national policy on this regard; thus, salt and salty seasonings added to meals from sit-down restaurants were not taken into account in this analysis. Ingredients of these dishes were considered separately for food record analysis, except salt and salty seasonings, due to the complexity of accurately estimating the amount of salt and salty seasonings used in meal preparation. Initiatives to promote reporting on nutrient levels of restaurant foods are needed, to better inform the population on sodium content in foods eaten outside the home, and to help individuals make healthier choices.

Fast foods such as pizzas was found among the top 10 food sources of dietary sodium, and they were the second with the highest percent of total sodium intake among consumers. Local street-foods such as tacos and tamales were also identified as relevant food contributors for total sodium intake in this population; indeed, tacos and tamales were first and the third with the highest percent of total sodium intake among consumers. However, it is important to mention that other Mexican street foods such as sopes (fried corn tortilla topped with refried beans, fresh cheese, and salsa) and gorditas (snack food made of corn dough and filled with pork skin, fresh cheese, and salsa), when both combined, were found to be consumed by 8% of this population, but were not included in the analysis as a whole food, since there was no available information on their sodium content in the nutrient software database, or in the tables of nutrient composition of Mexican foods, and similar to restaurant meals, the ingredients of these Mexican street foods, except table salt, were considered separately for analysis.

### Study Limitations

This study has three main limitations: (1) This analysis included foods for which sodium content was reported either in the nutrient software database, tables of nutrient composition of Mexican foods, or on food labels, possibly excluding relevant foods sources of sodium in the Mexican population for which there was no available information on sodium levels, such as restaurant dishes, and some Mexican street foods; (2) criteria to identified food sources of dietary sodium to be included in the analysis captured foods with a sodium density >120 mg sodium/100 g, which assured inclusion of moderate sodium content foods, but excluded low sodium density foods such as milk (approx. 44 mg of sodium in 100 g), that might represent certain percentages of total sodium intake in this population, especially when these low sodium foods are highly consumed by the population. Thus, the method used in this study to identify relevant food sources of sodium may have left out some other relevant sources of sodium that may explain, in part, the remaining 47% of total sodium intake not explained by the 26 food categories included in this analysis. However, we were able to identified processed foods with salt added and bread products (packaged and unpackaged) as main sources of dietary sodium intake and the main objective of product reformulation initiatives; (3) this study population included healthy volunteer workers from a health institution who enrolled in the study after attending an informative session on sodium intake and its role in human health, and thus they may be not comparable to the general population; additionally, these workers had access to a lunch service benefit (the institution provides lunch for free to all the personnel), thus eating patterns may not reflect sources of sodium in all segments of the Mexican population; (4) finally, the three-day food record method may have incorporated reporting bias to the dietary sodium estimates; however, in order to minimize this bias, all food records were reviewed by the study dietitian during an interview with the patient to identify any missing food items, and to clarify food item descriptions and portion sizes using standardized food models. Additionally, this dietary method included three days allowance to account for day-to-day variations in the eating patterns.

## 5. Conclusions

Overall, mean daily sodium intake estimated by three-day food records and 24-h urinary sodium excretion in this sample of the Mexican SALMEX cohort was 2647.2 mg/day and 3497.2 mg/day, respectively, which was higher than the 2000 mg/day intake recommended by the WHO. Bread products (savory bread and sweet bakery), processed meats, and cheeses were the top three contributors for total sodium intake, as measured by three-day food records in this study population, highlighting the need for public health policies focused on reducing the sodium content of processed food. Additionally, Mexican street foods such as tacos and tamales were found to be relevant food sources of sodium in this population. This data will raise awareness about the need for sensitizing the public about the contribution of these unpackaged foods (which have no food labels showing sodium levels) to total sodium intake. Also, it is important to set voluntary or regulated initiatives to report sodium levels in restaurant dishes that allow the public to make informed choices when eating out. Finally, further studies aimed at evaluating the contribution of table salt added during cooking in this population, and studies to establish sodium levels in diverse Mexican street foods are needed, to better understand the contribution of local eating patterns to the total dietary sodium intake in the Mexican population.

## Figures and Tables

**Table 1:** Study population characteristics by sex ^1^.

Variable	Overall (*n* = 950)	Men (*n* = 358)	Women (*n* = 592)	*p* Value ^2^
Age (years)	38.6 ± 10.7	37.6 ± 10.8	39.1 ± 10.6	0.04
Body mass index (kg/m^2^)	27.1 ± 4.8	27.3 ± 4.3	27.0 ± 5.0	0.30
Systolic blood pressure (mmHg) ^3^	119.0 ± 14.0	125.2 ± 13.7	115.4 ± 12.8	<0.001
Diastolic blood pressure (mmHg) ^3^	75.0 ± 9.3	77.5 ± 9.9	73.5 ± 8.6	<0.001
Dietary sodium intake (mg/day)	2647.2 ± 976.9	3018.2 ± 1091.9	2422.9 ± 823.6	<0.001
24-h urinary sodium excretion (mg/24 h) ^4^	3497.2 ± 1393.0	4167.7 ± 1520.2	3118.3 ± 1156.4	<0.001

^1^ Data are presented in mean ± standard deviation; ^2^ For comparison between men and women by using Student’s *t*-test for independent samples; ^3^ Systolic and diastolic blood pressure measures were available for 885 of the 950 included participants (men *n* = 330, women *n* = 555); ^4^ Only subjects that provided a complete 24-h urine sample were considered for estimating 24-h urinary sodium excretion (overall *n* = 698, men *n* = 252, women *n* = 446).

**Table 2:** Population consuming at least one food of each food group (consumers) by sex ^1^.

Food Category	Overall (*n* = 950)	Men (*n* = 358)	Women (*n* = 592)	*p* Value ^2^
Savory bread (bolillo, telera, baguette, sliced bread, hot dog buns, and others)	800 (84.2)	306 (85.5)	494 (83.4)	0.41
Processed meat (deli meat, sausages, bacon, chorizo, machaca and smoked pork chop)	692 (72.8)	268 (74.9)	424 (71.6)	0.28
Natural cheeses (Panela, Oaxaca, Goat, Parmesan, Brie, Camembert, Cheddar, Swiss, Gouda, Manchego, cream cheese, Provolone, etc.)	669 (70.4)	228 (63.7)	441 (74.5)	<0.001
Sweet bakery goods (packaged and unpackaged)	650 (68.4)	249 (69.6)	401 (67.7)	0.56
Salad dressings and mayonnaise	452 (47.6)	167 (46.6)	285 (48.1)	0.66
Cookies and cereal bars	369 (38.8)	125 (34.9)	244 (41.2)	0.054
Chips (potato, corn and wheat)	269 (28.3)	96 (26.8)	173 (29.2)	0.43
Tacos (assorted)	225 (23.7)	106 (29.6)	119 (20.1)	0.001
Breakfast cereal	206 (21.7)	60 (16.8)	146 (24.7)	0.004
Canned peppers (pickled and chipotle peppers)	176 (18.5)	67 (18.7)	109 (18.4)	0.91
Catsup and mustard	162 (17.1)	62 (17.3)	100 (16.9)	0.87
Tamales (assorted)	129 (13.6)	50 (14.0)	79 (13.3)	0.79
Margarine and butter	123 (12.9)	37 (10.3)	86 (14.5)	0.06
Mole	109 (11.5)	45 (12.6)	64 (10.8)	0.41
Canned fish (tuna and sardines)	104 (10.9)	39 (10.9)	65 (11.0)	0.97
Popcorn	99 (10.4)	29 (8.1)	70 (11.8)	0.07
Salty nuts and seeds	97 (10.2)	31 (8.7)	66 (11.1)	0.22
Wheat flour tortillas	96 (10.1)	38 (10.6)	58 (9.8)	0.69
Hot sauces and chamoy (bottled snack sauces)	91 (9.6)	25 (7.0)	66 (11.1)	0.04
Seasonings (seasoning sauces, salty seasoning powders and broth cubes)	84 (8.8)	32 (8.9)	52 (8.8)	0.94
Pizza	75 (7.9)	31 (8.7)	44 (7.4)	0.50
Crackers	72 (7.6)	24 (6.7)	48 (8.1)	0.43
Processed cheese ^3^	69 (7.3)	29 (8.1)	40 (6.8)	0.44
Hamburgers	63 (6.6)	32 (8.9)	31 (5.2)	0.03
Hot chili powder seasonings	53 (5.6)	10 (2.8)	43 (7.3)	0.004
Canned beans	47 (4.9)	21 (5.9)	26 (4.4)	0.31
Chicken nuggets	21 (2.2)	9 (2.5)	12 (2.0)	0.62
Instant soups	13 (1.4)	5 (1.4)	8 (1.4)	1.0
Chocolate milk powder	12 (1.3)	5 (1.4)	7 (1.2)	0.77
Olives	7 (0.7)	4 (1.1)	3 (0.5)	0.44
Powdered milk	6 (0.6)	1 (0.3)	5 (0.8)	0.42
Hot cakes flour mix	6 (0.6)	1 (0.3)	5 (0.8)	0.42
Peanut butter	1 (0.1)	0 (0.0)	1 (0.2)	1.0

^1^ Data are presented as *n* (%); ^2^ For comparison between men and women by using Pearson chi-square test or Fisher’s exact test; ^3^ Processed cheese category included cheese products made from an emulsified blend of natural cheese. Includes spreads, blocks, and slices with or without added ingredients; e.g., American style cheese, spread, or melting slices.

**Table 3:** Total sodium contributed by the most frequently consumed food categories in the entire sample population (per capita) ^1,2^.

Food Category	Mg/Na/Day (*n* = 950)	% of Total Na Intake ^3^ (*n* = 950)
Processed meat (deli meat, sausages, bacon, chorizo, machaca and smoked pork chop)	223.4 (205.5, 241.3)	8.3 (7.6, 8.9)
Savory bread (bolillo, telera, baguette, sliced bread, hot dog buns, and others)	209.4 (198.2, 220.6)	8.3 (7.8, 8.7)
Sweet bakery goods (packaged and unpackaged)	178.2 (165.9, 190.4)	7.6 (7.0, 8.2)
Natural cheeses (Panela, Oaxaca, Goat, Parmesan, Brie, Camembert, Cheddar, Swiss, Gouda, Manchego, cream cheese, Provolone, etc.)	118.0 (108.5, 127.5)	5.0 (4.5, 5.4)
Tacos (assorted)	113.8 (89.2, 138.3)	5.2 (3.9, 6.5)
Breakfast cereal	52.7 (44.1, 61.2)	2.1 (1.7, 2.5)
Pizza	46.0 (33.2, 58.7)	1.6 (1.2, 2.1)
Tamales (assorted)	39.6 (32.3, 46.8)	1.8 (1.4, 2.3)
Chips (potato, corn and wheat)	31.8 (26.9, 36.6)	1.2 (1.0, 1.4)
Seasonings (seasoning sauces, salty seasoning powders and broth cubes)	31.5 (21.9, 41.0)	1.1 (0.8, 1.5)
Cookies and cereal bars	26.4 (22.6, 30.3)	1.1 (0.9, 1.3)
Canned fish (tuna and sardines)	26.3 (20.2, 32.3)	1.1 (0.8, 1.3)
Canned peppers (pickled and chipotle peppers)	25.2 (19.5, 30.9)	1.0 (0.7, 1.2)
Mole	24.9 (19.0, 30.8)	1.0 (0.7, 1.2)
Hamburger	22.6 (16.6, 28.5)	0.8 (0.6, 1.0)
Salad dressings and mayonnaise	20.5 (18.1, 23.0)	0.8 (0.7, 0.9)
Hot chili powder seasonings	19.5 (8.5, 30.6)	0.7 (0.3, 1.2)
Catsup and mustard	16.4 (13.0, 19.9)	0.6 (0.5, 0.7)
Wheat flour tortillas	15.9 (12.5, 19.4)	0.7 (0.5, 0.8)
Canned beans	12.7 (7.6, 17.7)	0.4 (0.3, 0.6)
Hot sauces and chamoy (bottled snack sauces)	12.7 (8.5, 16.8)	0.5 (0.3, 0.7)
Salty nuts and seeds	11.6 (7.2, 16.1)	0.4 (0.3, 0.6)
Processed cheese ^4^	10.7 (7.9, 13.5)	0.4 (0.3, 0.5)
Crackers	9.9 (6.6, 13.2)	0.4 (0.3, 0.5)
Popcorn	8.4 (5.7, 11.1)	0.4 (0.3, 0.5)
Margarine and butter	5.5 (4.1, 7.0)	0.2 (0.2, 0.3)

^1^ Data are presented as mean (95% CI); ^2^ Estimates are provided for food groups with a consumer prevalence greater than 5%; ^3^ Based on three-day food records; ^4^ Processed cheese category included cheese products made from an emulsified blend of natural cheese. Includes spreads, blocks, and slices with or without added ingredients; e.g., American style cheese, spread or melting slices.

**Table 4:** Total sodium contributed by most frequently consumed food categories among individuals who consumed the foods (consumers) ^1,2^.

Food Category	Mg/Na/Day	% of Total Na Intake ^3^
Pizza	582.1 (480.5, 683.7)	20.6 (17.2, 24.0)
Tacos (assorted)	480.2 (391.8, 568.7)	21.8 (16.9, 26.8)
Seasonings (seasoning sauces, salty seasoning powders and broth cubes)	355.8 (274.1, 437.5)	12.7 (9.6, 15.7)
Hot chili powder seasonings	350.3 (170.2, 530.4)	11.9 (6.0, 17.8)
Hamburgers	340.4 (301.9, 378.9)	12.3 (10.6, 13.9)
Processed meat (deli meat, sausages, bacon, chorizo, machaca and smoked pork chop)	306.7 (285.1, 328.2)	11.3 (10.6, 12.1)
Tamales (assorted)	291.5 (265.6, 317.4)	13.5 (10.9, 16.1)
Sweet bakery goods (packaged and unpackaged)	260.4 (246.5, 274.3)	11.1 (10.4, 11.9)
Canned beans	255.6 (180.1, 331.1)	8.4 (6.0, 10.8)
Breakfast cereal	243.0 (216.5, 269.4)	9.7 (8.5, 10.8)
Savory bread (bolillo, telera, baguette, sliced bread, hot dog buns, and others)	248.6 (237.2, 260.0)	9.8 (9.4, 10.3)
Canned fish (tuna and sardines)	240.0 (205.5, 274.4)	9.6 (7.9, 11.3)
Mole	216.7 (181.7, 251.7)	8.5 (6.7, 10.3)
Natural cheeses (Panela, Oaxaca, Goat, Parmesan, Brie, Camembert, Cheddar, Swiss, Gouda, Manchego, cream cheese, Provolone, etc.)	167.6 (156.0, 179.1)	7.0 (6.5, 7.6)
Wheat flour tortillas	157.8 (140.8, 174.7)	6.7 (5.7, 7.7)
Processed cheese ^4^	147.5 (128.3, 166.6)	5.4 (4.7, 6.2)
Canned peppers (pickled and chipotle peppers)	135.8 (110.8, 160.9)	5.2 (4.1, 6.4)
Crackers	130.8 (98.2, 163.3)	4.6 (3.5, 5.8)
Hot sauces and chamoy (bottled snack sauces)	132.0 (96.7, 167.4)	5.1 (3.6, 6.5)
Salty nuts and seeds	113.7 (75.3, 152.2)	4.1 (3.0, 5.3)
Chips (potato, corn and wheat)	112.1 (99.1, 125.1)	4.4 (3.8, 4.9)
Catsup and mustard	96.3 (81.1, 111.5)	3.3 (2.8, 3.8)
Popcorn	80.6 (59.2, 102.0)	3.5 (2.5, 4.5)
Cookies and cereal bars	68.1 (59.8, 76.3)	2.9 (2.5, 3.3)
Salad dressings and mayonnaise	43.1 (38.8, 47.4)	1.6 (1.5, 1.8)
Margarine and butter	42.7 (34.2, 51.3)	1.9 (1.3, 2.4)

^1^ Data are presented as mean (95% CI); ^2^ Estimates are provided for food groups with a consumer prevalence greater than 5%; ^3^ Based on three-day food records; ^4^ Processed cheese category included cheese products made from an emulsified blend of natural cheese. Includes spreads, blocks, and slices with or without added ingredients; e.g., American style cheese, spread or melting slices.

## References

[1] Farquhar W.B., Edwards D.G., Jurkovitz C.T., Weintraub W.S. (2015). Dietary sodium and health: More than just blood pressure. J. Am. Coll. Cardiol..

[2] Aburto N.J., Ziolkovska A., Hooper L., Elliott P., Cappuccio F.P., Meerpohl J.J. (2013). Effect of lower sodium intake on health: Systematic review and meta-analyses. BMJ.

[3] Graudal N.A., Hubeck-Graudal T., Jurgens G. (2011). Effects of low sodium diet versus high sodium diet on blood pressure, renin, aldosterone, catecholamines, cholesterol, and triglyceride. Cochrane Database Syst. Rev..

[4] He F.J., MacGregor G.A. (2002). Effect of modest salt reduction on blood pressure: A meta-analysis of randomized trials. Implications for public health. J. Hum. Hypertens..

[5] He J., Gu D., Chen J., Jaquish C.E., Rao D.C., Hixson J.E., Chen J.C., Duan X., Huang J.F., Chen C.S. (2009). Gender difference in blood pressure responses to dietary sodium intervention in the GenSalt study. J. Hypertens..

[6] He F.J., Li J., MacGregor G.A. (2013). Effect of longer term modest salt reduction on blood pressure: Cochrane systematic review and meta-analysis of randomised trials. BMJ.

[7] Vollmer W.M., Sacks F.M., Ard J., Appel L.J., Bray G.A., Simons-Morton D.G., Conlin P.R., Svetkey L.P., Erlinger T.P., Moore T.J. (2001). Effects of diet and sodium intake on blood pressure: Subgroup analysis of the DASH-sodium trial. Ann. Intern. Med..

[8] GBD 2015 Risk Factors Collaborators (2016). Global, regional, and national comparative risk assessment of 79 behavioural, environmental and occupational, and metabolic risks or clusters of risks, 1990–2015: A systematic analysis for the Global Burden of Disease Study 2015. Lancet.

[9] Campos-Nonato I., Hernández-Barrera L., Rojas-Martínez R., Pedroza A., Medina-García C., Barquera-Cervera S. (2013). Hypertension: Prevalence, early diagnosis, control and trends in Mexican adults. Salud Publica Mex..

[10] Vallejo M., Colin-Ramirez E., Rivera S., Cartas R., Madero M., Infante O., Vargas-Barron J. (2017). Assessment of sodium and potassium intake by 24-hour urinary excretion in a healthy Mexican population: The Tlalpan 2020 Cohort. Arch. Med. Res..

[11] World Health Organization (2014). Global Status Report on Noncommunicable Diseases 2014.

[12] World Health Organization (2012). Report of the Formal Meeting of Member States to Conclude the Work on the Comprehensive Global Monitoring Framework, Including Indicators, and a Set of Voluntary Global Targets for the Prevention and Control of Noncommunicable Diseases.

[13] Mattes R.D., Donnelly D. (1991). Relative contributions of dietary sodium sources. J. Am. Coll. Nutr..

[14] Ravi S., Bermudez O.I., Harivanzan V., Kenneth Chui K.H., Vasudevan P., Must A., Thanikachalam S., Thanikachalam M. (2016). Sodium intake, blood pressure, and dietary sources of sodium in an adult south indian population. Ann. Glob. Health.

[15] Zhao F., Zhang P., Zhang L., Niu W., Gao J., Lu L., Liu C., Gao X. (2015). Consumption and sources of dietary salt in family members in Beijing. Nutrients.

[16] Asakura K., Uechi K., Masayasu S., Sasaki S. (2016). Sodium sources in the Japanese diet: Difference between generations and sexes. Public Health Nutr..

[17] Anderson C.A., Appel L.J., Okuda N., Brown I.J., Chan Q., Zhao L., Ueshima H., Kesteloot H., Miura K., Curb J.D. (2010). Dietary sources of sodium in China, Japan, the United Kingdom, and the United States, women and men aged 40 to 59 years: The INTERMAP study. J. Am. Diet. Assoc..

[18] Ferrante D., Apro N., Ferreira V., Virgolini M., Aguilar V., Sosa M., Perel P., Casas J. (2011). Feasibility of salt reduction in processed foods in Argentina. Rev. Panam. Salud Publica.

[19] Vega O., Mendoza A., Baeza Y., Rincón R., Espinosa-Cuevas A., Fonseca J., Nieves I., Herrero B., Correo R. (2012). Asociación de la ingesta dietética de sal con hipertensión en trabajadores mexicanos: Estudio SALMEX. Conference Abstract Booklet, Proceedings of the LXI Annual Meeting of the Mexican Institute of Nephrology Research (Instituto Mexicano de Investigaciones Nefrológicas (IMIN)), Guadalajara, Mexico, 5–8 December 2012.

[20] Centers for Disease Control and Prevention, National Center for Health Statistics (2007). National Health and Nutrition Examination Survey (NHANES).

[21] Morales de León J.C., Bourges Rodríguez H., Camacho Parra M.E. (2016). Tablas de Composición de Alimentos y Productos Alimenticios Mexicanos (Versión Condesada 2015).

[22] Rhee J.J., Sampson L., Cho E., Hughes M.D., Hu F.B., Willett W.C. (2015). Comparison of methods to account for implausible reporting of energy intake in epidemiologic studies. Am. J. Epidemiol..

[23] Yum J., Lee S. (2016). Development and evaluation of a dish-based semiquantitative food frequency questionnaire for Korean adolescents. Nutr. Res. Pract..

[24] Teixeira J.A., Baggio M.L., Giuliano A.R., Fisberg R.M., Marchioni D.M. (2011). Performance of the quantitative food frequency questionnaire used in the Brazilian center of the prospective study natural history of human papillomavirus infection in men: The HIM study. J. Am. Diet. Assoc..

[25] U.S. Food and Drug Administration (2016). How to Understand and Use the Nutrition Facts Label.

[26] Health Canada, Bureau of Nutritional Sciences (2012). Guidance for the Food Industry on Reducing Sodium in Processed Foods.

[27] Pelleg A., Levy G.B. (1975). Determination of Na+ and K+ in urine with ion-selective electrodes in an automated analyzer. Clin. Chem..

[28] Wielgosz A., Robinson C., Mao Y., Jiang Y., Campbell N.R., Muthuri S., Morrison H. (2016). The impact of using different methods to assess completeness of 24-hour urine collection on estimating dietary sodium. J. Clin. Hypertens. (Greenwich).

[29] Carballo de la Espriella M., Morales Palma G. (2011). Fuentes Alimentarias de sal/sodio en mujeres, Costa Rica. Rev. Costarric. Salud Pública.

[30] Gaitan Charry D.A., Estrada A., Argenor L.G., Manjarres L.M. (2015). Food sources of sodium: Analysis based on a national survey in Colombia. Nutr. Hosp..

[31] Meneton P., Lafay L., Tard A., Dufour A., Ireland J., Menard J., Volatier J.L. (2009). Dietary sources and correlates of sodium and potassium intakes in the French general population. Eur. J. Clin. Nutr..

[32] Fischer P.W., Vigneault M., Huang R., Arvaniti K., Roach P. (2009). Sodium food sources in the Canadian diet. Appl. Physiol. Nutr. Metab..

[33] De Moura S.A., Bezerra I.N., Pereira R.A., Peterson K.E., Sichieri R. (2013). Dietary sources of sodium intake in Brazil in 2008–2009. J. Acad. Nutr. Diet..

[34] Campbell N., Legowski B., Legetic B., Ferrante D., Nilson E., Campbell C., L’Abbe M. (2014). Targets and timelines for reducing salt in processed food in the Americas. J. Clin. Hypertens. (Greenwich).

[35] Campbell N. (2015). Population Level Dietary Salt Reduction Initiative in the Americas. http://resources.cpha.ca/CPHA/Conf/Data/2015/A15-633e.pdf.

[36] Secretaría de Gobernación (2012). Acuerdo Por el Que se Recomienda la Disminución del Uso de Sal Común o Cloruro de Sodio en la Elaboración de Pan Como Una Medida de Prevención de Enfermedades Cardiovasculares, y Otras Crónico-Degenerativas. http://dof.gob.mx/nota_detalle.php?codigo=5256201&fecha=22/06/2012.

[37] García Urigüen P. (2012). La Alimentación de los Mexicanos. Cambios Sociales y Económicos, y su Impacto en Los Hábitos Alimenticios.

